# RichMind: A Tool for Improved Inference from Large-Scale Neuroimaging Results

**DOI:** 10.1371/journal.pone.0159643

**Published:** 2016-07-25

**Authors:** Adi Maron-Katz, David Amar, Eti Ben Simon, Talma Hendler, Ron Shamir

**Affiliations:** 1 Functional Brain Center, Wohl Institute for Advanced Imaging Tel-Aviv Sourasky Medical Center, Tel Aviv, Israel; 2 Sackler Faculty of Medicine, Tel Aviv University, Tel Aviv, Israel; 3 Blavatnik School of Computer Science, Tel-Aviv University, Tel Aviv 69978, Israel; 4 School of Psychological Sciences, Tel Aviv University, Tel-Aviv 69978, Israel; 5 Sagol school of Neuroscience, Tel Aviv University, Tel Aviv, Israel; University of Texas at Austin, UNITED STATES

## Abstract

As the use of large-scale data-driven analysis becomes increasingly common, the need for robust methods for interpreting a large number of results increases. To date, neuroimaging attempts to interpret large-scale activity or connectivity results often turn to existing neural mapping based on previous literature. In case of a large number of results, manual selection or percent of overlap with existing maps is frequently used to facilitate interpretation, often without a clear statistical justification. Such methodology holds the risk of reporting false positive results and overlooking additional results. Here, we propose using *enrichment analysis* for improving the interpretation of large-scale neuroimaging results. We focus on two possible cases: *position group analysis*, where the identified results are a set of neural positions; and *connection group analysis*, where the identified results are a set of neural position-pairs (i.e. neural connections). We explore different models for detecting significant overrepresentation of known functional brain annotations using simulated and real data. We implemented our methods in a tool called RichMind, which provides both statistical significance reports and brain visualization. We demonstrate the abilities of RichMind by revisiting two previous fMRI studies. In both studies RichMind automatically highlighted most of the findings that were reported in the original studies as well as several additional findings that were overlooked. Hence, RichMind is a valuable new tool for rigorous inference from neuroimaging results.

## Introduction

To date, functional interpretation of large-scale neuroimaging findings is often done by associating the identified regions to known *classes* (e.g., anatomic structures or functional networks). This process of using previous knowledge to ascribe functional meaning to findings is commonly based on a subjective visual inspection or on percent of overlap with existing maps [1–5]. Such methodology, which is not based on statistical justification, holds the risk of reporting false positive results and overlooking additional results. For example, Nummenmaa et al. (2012) analyzed fMRI signals recorded from 16 healthy participants, while viewing film clips depicting unpleasant, neutral, and pleasant emotions. They identified cerebral regions where inter-subject correlations (ISC) were significantly correlated with subjective reports of valence and arousal provided by the participants. In order to interpret the findings, the authors subjectively associated the identified regions to known functional networks. They reported that arousal was mostly associated with ISC in regions of the sensori-motor network (SMN), visual network (VN) and dorsal attention network (DAN) while valence was negatively associated with ISC in regions of the default mode network (DMN) as well as regions known to be involved in emotional processing [2]. However, as in many neuroimaging studies, no quantitative statistical measure was presented to support this association of findings to functional networks. An even more complex case is the case where the identified findings are a collection of neural position pairs (i.e. connections). When this collection is very large, as may occur in data-driven studies [6–8], interpretation becomes challenging. In some cases, this challenge is faced by filtering the results either by manual selection or by repeating the analysis using a stricter statistical threshold. For example Wang et. al. (2013) reported a set of 363 functional connections that differed between a group of amnestic mild cognitive impairment (aMCI) patients and healthy controls. These connections were identified using the network-based statistic approach [9] with a predefined 1024 functional parcellation [1, 10]. In order to avoid the complexity of interpreting such a large set of connections, the network-based statistic analysis was repeated using a stricter statistical threshold, producing a set of 87 connections that was subsequently interpreted.

An alternative approach to interpreting such a large set of findings is to test whether the results contain significantly more elements with a specific class than expected by chance. For instance, one can examine whether an identified set of weakened connections in terms of DMN-SMN connectivity, and explore whether aMCI is associated with a significantly large number of weakened connections linking the DMN with the SMN. If the answer is positive, we say that the corresponding class (i.e. DMN-SMN) is *enriched* in the identified collection. Such enrichment (or over-representation) can be assigned with a statistical significance value under an appropriate null hypothesis [11].

In this study we propose using *enrichment analysis* to facilitate and improve the interpretation process of large-scale results obtained from fMRI studies. Notably, the term *enrichment analysis* in this case refers to the analysis of significance of class overrepresentation within sets (i.e. overlap) [11–13], and not to the analysis of significance of class distribution across a ranked set [14]. We address two cases: In the first case the identified results are a set of neural positions. For example, these positions could be a set of voxels that demonstrate increased activation under a specific condition. In this case, we call the enrichment analysis *position group analysis*. In the second case, the identified results are a set of neural position pairs (i.e. neural connections). For example, they can be pairs of neural positions demonstrating increased functional connectivity under a specific experimental condition [6–8]. In this case, we call the enrichment analysis *connection group analysis*. In both cases, in addition to the study results we are given an annotation of the brain that maps neural positions to classes representing known neural functions (e.g. [15–17]), or anatomic structures (e.g. [18–20]). We examined different models for detecting significant overrepresentation of known functional brain annotation using simulated and real data.

We implemented our methods in RichMind, an open-source Matlab-based computational tool that provides both statistical significance reports based on our suggested enrichment analysis methods, as well as brain visualizations. We demonstrate the abilities of RichMind by reanalyzing two previous fMRI studies: the first of subjects viewing emotion-inducing film clips [2], and the second of subjects suffering from aMCI [1]. We show that by using enrichment analysis, we were able to provide statistical validation to most of the conclusions drawn in the original studies, while revealing additional statistically significant results. In addition we show how enrichment analysis allows interpreting a large set of results without having to apply additional filters, as often applied in studies, thus, allowing more accurate interpretation of the results.

## Methods

### Neural annotation

A *neural annotation* is a mapping of neural positions to known classes. The annotation is based on previous knowledge, and can contain anatomic structure (E.g. Anatomic atlas labeling [21]), known functional mapping (E.g. functional networks identified in previous studies [15, 22]), previously known pathology association, etc.

In the current study we used two sets of annotations that are based on functional neural mapping. The first was used in [2] and it consists of 6 functional networks, and the second annotation was used in [1] and it consists of 5 functional brain networks. In our simulation, we used a made-up dummy annotation.

### The Hypergeometric test

**Fig 1:**
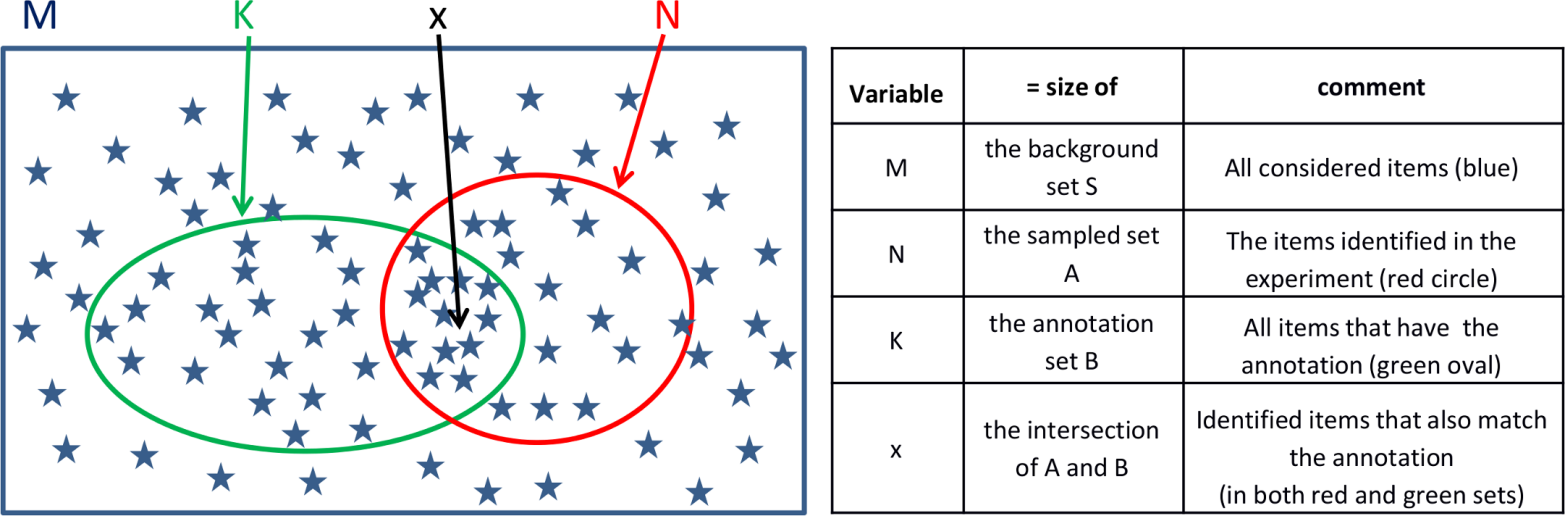
**A demonstration of HG test setting**: given a background set S, which contains M elements (starts), a specific annotation set B with K elements, and a randomly sampled set A with N elements, HG test estimates the probability of having x or more elements of B in A.

In this study, we use the hypergeometric (HG) test to calculate the significance of the overlap of two sets. The test is demonstrated in Fig 1. We are given a set *S* of *M* items, which we call the *background set*, and two subsets *A* ⊆ *S* and *B* ⊆ *S*, of sizes *N* and *K*, respectively. (For example, S can be the set of brain positions considered, *A* is the set of positions identified in the experiment, and B is a particular subset of the positions annotated with a known structure or function, e.g. the cerebellum.) Let *x* be the size of the intersection between *A* and *B*. Is that intersection size meaningful or random? Here, our null hypothesis is that the N items in A were sampled randomly and independently from S without replacement. Therefore, the significance of the observed value x is the probability of having x or more elements in the intersection, which can be calculated using the HG cumulative distribution function, given in Eq 1. Notably, the Hypergeometric test is equivalent to a one-tailed Fisher’s exact test. Equation variables are listed in Fig 1.

$$p=F(x|M,K,N)=\sum_{i=x}^{min(N,K)}\frac{\left(\begin{array}{c} K\\ i \end{array})(\begin{array}{c} M-K\\ N-i \end{array}\right)}{\left(\begin{array}{c} M\\ N \end{array}\right)}$$ (1)

### Position group enrichment analysis using the HG test

Here, the study identified a group of neural positions (e.g., voxels or parcels) A^p^ out of a background set S. We wish to consider the significance of the overlap of A^p^ with another subset of neural positions *B* from S, which constitutes a known annotation class (e.g., an anatomically defined brain region or a set of regions that share a specific function or form a particular network). Our goal is to decide whether the number x of positions that are both in group A^p^ and class B is larger than expected by chance.

### Connections group enrichment analysis

Here the study identified a group of connections (i.e. pairs of neural positions) A^c^ = {(x_1_,y_1_),…,(x_n_,y_n_)}, where each x_i_ and each y_i_ represents a neural position (e.g. voxel or parcel). This set can also be viewed as the set of edges in a graph G(S, A^c^), where S is the set of neural positions. In addition we are given two disjoint subsets of S, C and D, each of which constitutes a known annotation class (e.g. C is the precuneus and D is the basal ganglia). Our goal is to decide whether the number of connections between C and D that are also in A^c^, denoted as a(A^c^,C,D), is larger than expected by chance. In this work, we test two approaches for this task: (1) a parametric approach that uses the HG test; and (2) a non-parametric test based on permutations. While the first test is relatively simple and easy to compute, it ignores the degree distribution in the graph represented by the neural connections. This is addressed by the second test, which uses permutations to create a large set of random graphs with the same node degrees. These two tests have been validated on simulated data (See S1, S2 and S3 Figs), and the difference between them is illustrated in S1 and S4 Figs. In the next two sections we use the same notation as above.

### Parametric connection group analysis using the HG test

Here, we use the HG test with the following parameters. N is the number of pairs in A^c^. M (i.e., the background set size) is the number of all possible neural pairs: M = |S|(|S|-1)/2. K = |C|*|D| is the number of possible pairs between C and D. Finally, x is the observed number of pairs between C and D—a(E,C,D). The underlying null hypothesis of this test is that the graph G was randomly selected from the set of all graphs with the same number of edges N over the same set of nodes.

### Non-parametric degree-preserving analysis

The HG approach for connection groups does not account for the degree distribution in the graph G. The importance of this distribution has been previously observed in brain networks [23, 24]. We therefore propose an additional non-parametric test. Here, our null hypothesis is that the graph G was randomly selected from the set Σ of all graphs with the same node degrees. In other words, Σ is the set of graphs with node set S such that each node has the same number of neighbors as in G.

We calculate the p-value empirically by drawing graphs from S using a heuristic rewiring step: remove two disjoint edges in the current graph and replace them by two others so that node degrees remain unchanged. A long chain of such steps leads to a near-random sampling from S [25]. The method has been successfully used in multiple bioinformatics applications [26, 27]. Given a set of graphs generated using this process we calculate for each one the number of observed edges between C and D. This step produces a vector of scores a = a_1_,…,a_m_ (by default we generate m = 1000 randomized graphs). The final empirical p-value is the fraction of scores in a greater than or equal to a(E,C,D). We call this process degree preserving permutation (DPP), as the edges in each random graph generated form a permutation of the original edges that also preserves the degree constraints.

The DPP and the HG tests use different null models and hence can differ in their sensitivity depending on the data. The HG test assumes that all possible graphs with N edges are equally likely to be selected, and the DPP test assumes that only graphs with N edges and with the same number of neighbors for each node are equally likely to be selected. As a result, the former is more sensitive when the number of identified connections between C and D is relatively high compared to M, while the latter is more sensitive when the identified connections between C and D is relatively high compared to the number of identified connections involving C or D. See S1 Text and S1 and S4 Figs for an example and simulations that demonstrate the difference.

### Multiple testing correction

Since enrichment is tested for each combination of an identified collection and a class, the output contains multiple p-values. Therefore, we correct for multiple testing at a false discovery rate (FDR) of 0.05 using the procedure suggested by Benjamini and Hochberg (BH) [28]. The FDR approach controls for the rate of false discovery, and is considered to be more powerful than methods that control for the family-wise error rate such as Bonferroni approach [28–30]. The FDR-adjusted p-value of each enrichment is called its q-value [31].

## Results

We implemented the two statistical tests for enrichment in results of fMRI studies in a Matlab-based tool called RichMind. Below, we first give a brief explanation on the input and output of the tool. Next, we show two case studies in which we apply RichMind to real fMRI data.

### RichMind–a toolbox for analysis of enrichment of fMRI results

RichMind receives as input a collection of items identified in a neuroimaging study. These can be either (1) one or more sets of neural positions for position group analysis, or (2) a set of neural position-pairs for connection group analysis. Each set of positions in (1) can be input using (a) a 3D NIFTI file, containing at each position a numerical group identifier (the number of the set to which that position belongs), (b) a tabular text file, in which each line contains a numerical position identifier followed by a numerical group identifier, or (c) a Matlab data file, in which each line contains a numerical position identifier followed by a numerical group identifier. Sets of position-pairs in (2) can be input using similar formats as (b) or (c) with three columns, where the first two columns contain position identifiers and the third column contains the group identifier. In addition to the groups file, the collection of all positions considered in the experiment is required. This collection is used to define the background set, and should preferably include all positions used in the initial analysis that yielded the interest groups. The file can be a 3D NIFTI file, a tabular text file or a Matlab file. In both cases each line should contain the MNI coordinates of a single position, and the line number is used as the position identifier in (1) and (2).

RichMind uses an established neural annotation, attributing neural positions to meaningful terms. These classes reflect prior knowledge of brain function or anatomic structure, so they can be anatomic labels, functions, pathology association, etc. By default, RichMind uses the functional neural mapping provided in [15] as the annotation. Alternatively, it provides an option to use the anatomic mapping provided in [32], or any other mapping provided by the user. In each type of analysis RichMind calculates the p-values for over-representation of the classes (see Methods for details). When using the DPP test, if *k* is the number of graphs sampled, then the minimum empirical p-value that can be obtained is 1/*k*. Thus, a larger *k* increases the maximum significance achievable, but also the test runtime. For example, in case study 2, running DPP with 1000 graphs gave a maximum significance of 10^−3^ and took 212 seconds on a quad core Pentium i7 processor, with 8Gb RAM. Since many groups are tested, the use of nominal p-values is inadequate and correction for multiple testing is needed. Hence, all p-values are corrected for multiple testing using the false discovery rate (FDR) method [28] and q-values are reported. Alternatively, the user can choose the more stringent Bonferroni correction.

**Fig 2:**
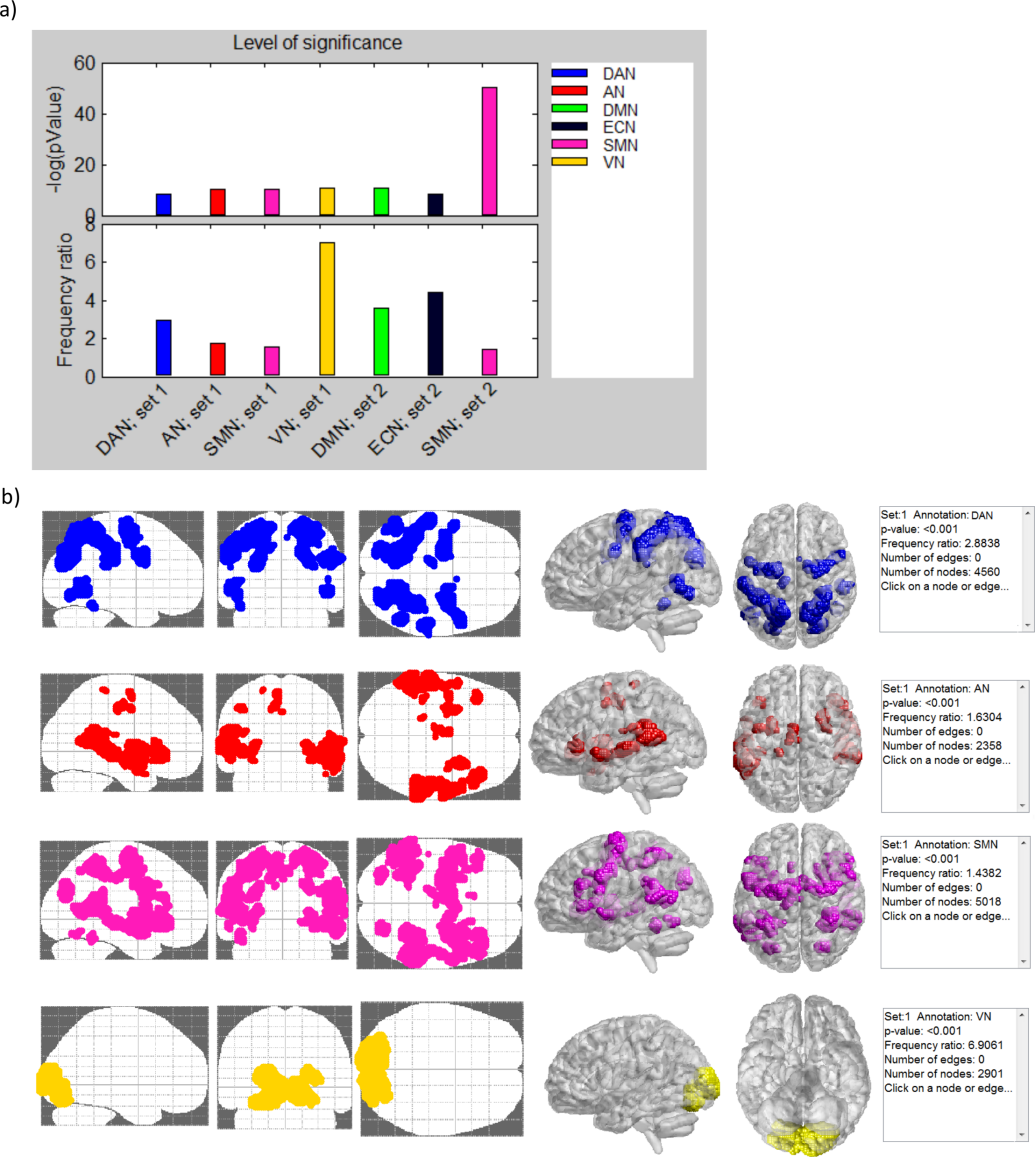
**RichMind results visualization for case study 1:** (a) Bar plots displaying the p-values and frequency ratios of enrichment analysis results. Each bar is colored according to the enriched class. (b) 2D and high-resolution 3D brain visualization, which shows, for each enriched class, all neural positions that are both in the SOI and in the class. Positions are colored according to the corresponding classes. High resolution 3D images were generated using BrainNet viewer [33].

**Fig 3:**
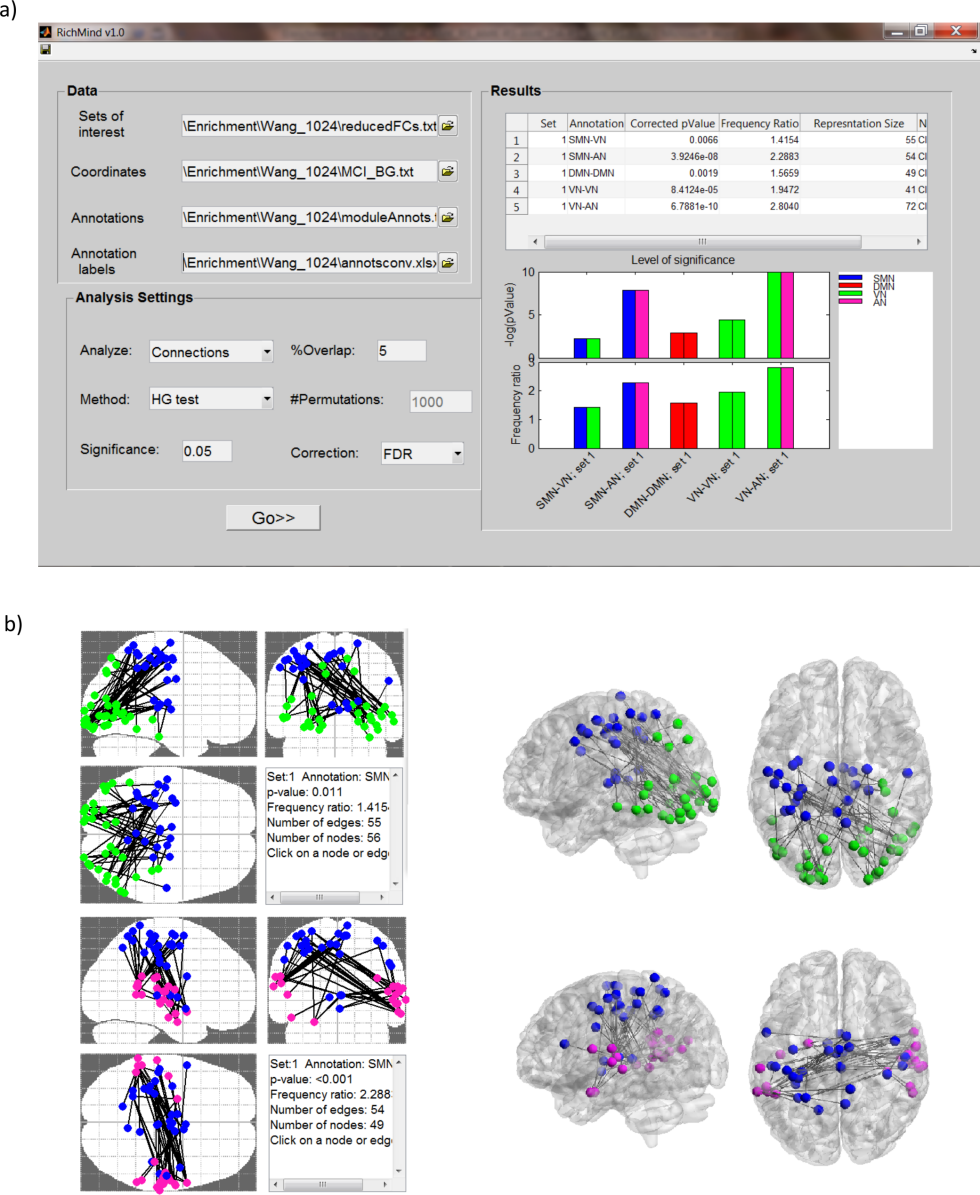
**RichMind results visualization for case study 2:** (a) Bar plots displaying the p-values and frequency ratios of enrichment analysis results. Each bar is composed of two rectangles colored by the two classes that constitute the enriched class. (b) 2D and high-resolution 3D brain visualization, showing, for each enriched class, all neural connections that are both in CC363 and in the class. Parcels are colored according to the corresponding classes. High resolution 3D images were generated using BrainNet viewer [33].

Finally, RichMind reports a list with all significant enrichments (0.05 FDR by default), and also produces bar plots that display the p-value and an additional measure of enrichment level called the “frequency ratio” result (see Figs 2A and 3A for examples). The frequency ratio is the ratio between fraction of class members in the tested set and in the background set. This measure evaluates how frequent is the class representation in the set vs. the background. High ratios indicate over-representation, but do not have direct statistical significance as p-values, and should be used cautiously when comparing results that involve classes of very different sizes. On the other hand, when class or sets are too small to provide significant p-values, high ratios can call attention to particular results. Ratios should be used cautiously when comparing results that involve classes of very different sizes. For each reported result, brain 2D and 3D views overlaying the neural positions (or connections) are available by clicking on the result (see Figs 2B and 3B for examples). In addition, one can export these overlay graphs into files that can be loaded to the BrainNet viewer [33] (see Figs 2B and 3B for examples).

### Case Study 1: Inter-subject correlation identified while watching emotion inducing film clips

Here we revisited findings reported by Nummenmaa et al. (2012), who identified ISC maps that were correlated with self-reported valence and arousal scores. ISCs were based on fMRI data recorded from 16 healthy participants, while viewing film clips depicting unpleasant, neutral, and pleasant emotions. ISCs were derived by calculating, for each voxel, the Pearson correlation coefficient of the BOLD time series recorded in each pair of subjects. This was done both for the entire time frame and for sliding windows of 17 time points. Ongoing measures of self-reported valence and arousal provided by participants were used as regressors in a general linear model (GLM), to identify significantly associated ISCs. Results were interpreted by the authors in the context of six functional networks extracted using seed-based functional connectivity analysis on the same data–the VN, SMN, AN, DMN, DAN and the executive control network (ECN). The authors reported that arousal was mostly associated with ISC in the SMN, VN and DAN while valence was negatively associated with ISC in the DMN as well as in regions involved in emotional processing, such as midbrain, thalamus, ventral striatum, insula, and anterior cingulate cortex [2]. No quantitative statistical measure was presented to support this interpretation.

We ran RichMind position group analysis on two sets of cerebral regions: one where ISC was inversely associated with self-reported valence, and another where ISC was positively associated with self-reported arousal. All gray matter voxels were used as background for enrichment test. The mapping of voxels to the six functional networks was taken from the original paper. The results are presented in Table 1 and Fig 2. RichMind identified arousal associated ISC to be enriched with regions involved in AN (q = 1.28E-10), SMN (q = 1.85E-10), DAN (q = 9.28E-09), and VN (q = 9.26E-11), and valence associated ISCs to be enriched with DMN (q = 9.26E-11), SMN (<1.4E-37) and ECN (q = 6.59E-09). These results recapitulate the results of the original paper. However, they add additional findings of AN enrichment within arousal associated ISCs, and ECN and SMN enrichment within valence associated ISCs. These findings reinforce the claim made in the original study, by which high arousal serves to direct individuals’ attention to features of the environment. Identifying ECN and SMN enrichment within valence associated ISCs, is in line with the authors’ suggestion by which negative valence synchronizes brain circuitries, supporting emotional sensations across individuals.

**Table 1:** RichMind results for case study 1. DAN = dorsal attention network, AN = auditory network, SMN = sensori-motor network, VN = visual network, DMN = default-mode network, ECN = executive control network.

Set	Enriched class	HG-based q-values	Frequency Ratio	# Voxels
Arousal ISCs	DAN	9.28E-09	2.9	4560
Arousal ISCs	AN	1.28E-10	1.6	2358
Arousal ISCs	SMN	1.85E-10	1.4	5018
Arousal ISCs	VN	9.26E-11	6.9	2901
Valence ICSs	DMN	9.26E-11	3.5	1357
Valence ICSs	ECN	6.59E-09	4.3	2684
Valence ICSs	SMN	<1.4E-37	1.3	2056

### Case Study 2: fMRI functional connectivity differences identified in cases of amnestic mild cognitive impairment

Wang et al. (2013) analyzed resting state fMRI data recorded from 37 subjects with aMCI, and 47 healthy controls. The analysis produced functional connections that differed between the groups. These connections were identified using the network-based statistic approach [9] on a predefined functional parcellation containing 1024 parcels [1, 10]. The approach identified connected components (CCs) that are composed of FCs for which the inter-group difference exceeded a pre-defined threshold. Component significance was estimated using a permutation test. This analysis detected a single CC of 363 reduced FCs when using a p-value threshold of 5*10^−4^. We call this set CC363. In addition, two CCs of 65 and 22 reduced FCs were discovered using a p-value threshold of 10^−4^, denoted as CC65 and CC22, respectively.

In the original study, due to the large number of connections in CC363, only CC65 and CC22 were further interpreted. This was done in the context of a modular architecture derived from the control group, which includes five modules corresponding to the VN, the SMN, the DMN, the AN and the ventral attention network (VAN). CC65 was reported as comprised mainly of inter-module connections (46/65, 70.8%), which linked regions in the SMN module, the VN module, and the AN module. CC22 was reported to contain predominantly intra-module connections (15/22, 68.2%) within the DMN module [1].

We used RichMind to analyze CC363. All 1024 parcels were used to generate the background for the enrichment analyses. A mapping of nodes to functional modules was taken from the original paper. The results are presented in Table 2 and Fig 3. The HG-based analysis identified CC363 as enriched with inter-modular FCs that link regions of the SMN module, the VN module, and the AN module (q_(SMN-VN)_ = 0.011, q_(SMN-AN)_ = 6.54E-08; q_(AN-VN)_ = 1.2E-09), and with intra-modular connections within the DMN module (q = 0.003). These results reproduce the main conclusions of the original study, but were obtained on the larger CC, which was not discussed in the original study due to its size. In addition, the test revealed enrichment in connections within the VN module (q = 0.00014), which was not reported in the original study. The degree preserving permutation test did not identify FC enrichment within the VN nor did it identify FC enrichment between the SMN and the VN. However, it recovered the other three inter-module links (see Table 2). Notably, the differences between HG and DPP in this case are primarily for connections that involve the VN. This is probably due to the relatively low degree of VN nodes in the CC363 component graph. While HG is not sensitive to the degree of nodes in each class, DPP does consider this information, and favors higher density links between classes (See also S4 Fig and S1 Text).

**Table 2:** RichMind results for case study 2; Class abbreviations: VN = visual network, AN = auditory network, SMN = sensori-motor network, DMN = default-mode network.

Enriched inter-class connection	HG q-value	DPP q-value	Frequency ratio	# Connections
VN-AN	6.8E-10	0.0325	2.8	72
SMN-AN	3.9E-08	0.00075	2.33	54
VN-VN	8.4E-05	0.79	1.9	41
DMN-DMN	0.0019	<0.00075	1.66	49
SMN-VN	0.0066	0.79	1.4	55

### Repeating case analyses with an external annotation

The above analyses were conducted using the same annotations that were used by the authors of the original papers for interpretation. In both cases, these were functional brain networks that were extracted from the same experiment. However, to validate the results of a new experiment, it is preferable that enrichment analysis is conducted using an independent annotation. Another advantage of using such an external annotation is that it allows the results to be comparable across studies. Accordingly, we repeated both case analyses using an annotation reported in [19], which includes a partition of the cortex into seven functional brain networks. This annotation was selected because it is based on a thorough analysis of a very large cohort of 1000 subjects. When we repeated the analysis of RichMind using this annotation two of the 5 results in our previous analysis of case study 2 were identified (VN-SMN and VN-VN connectivity). In case study 1, the results remained similar to those obtained in our previous analysis, see supporting S1 Table and S2 Table. However, slight differences were identified. For example, valence associated ISC was enriched with SMN using the original annotation but not the external one. This difference results from discrepancies in the mapping of voxels to networks.

## Discussion

In this work we describe RichMind, a Matlab-based, easy-to-use computational tool that tests for enrichment of known classes in large-scale neural results. It provides both statistical reports and brain visualizations of the identified enrichments. Statistical reports are based on the probability of getting the observed representation of each annotation in the tested set by chance.

We applied RichMind to two case studies, and in both of them RichMind reinforced the main claims made in the original papers, while adding new findings. In case study 1, the involvement of the ACC in the valence-associated ISCs seems to contribute most to the identified ECN enrichment within that group (Fig 2B, shown in black), in accordance with the statement in the original study. However, regions of the SMN were reported in the original study only in association with arousal ISCs and not with valence ISCs. Using RichMind we reveal SMN enrichment within valence associated ISCs, a finding with extremely low q-value, indicating that it is highly significant. Notably, this enrichment was not identified using the external annotation that was based on [15], due to differences between the mappings. This inconsistency demonstrates the need for an established functional mapping of the brain that is acknowledged in the field as “common ground”.

While enrichment analysis is standard in genomic and genetic studies [11, 12, 34], few previous fMRI studies addressed the issue of large-scale interpretation by calculating the relative frequency of specific classes. For example, in case study 2, Wang et al. [1] used maps of known functional brain networks extracted from the set of healthy controls through modularity analysis, and then calculated the percent of the results that link each pair of networks [1]. However, such approach does not take into consideration the spatial coverage of each class, which has a major effect on the frequency of its representation in the results. Furthermore, it does not provide statistical significance of the reported findings.

Unlike the simple case where results contain sets of neural positions, when examining sets of neural connections, the null hypothesis of random independent sampling, which underlies the hyper-geometric test, may not be suitable. This is due to a non-uniform distribution of the degrees in the brain network [23, 24]. Instead, empirical p-values can be calculated using a permutation test, in which the random background model preserves the degrees of the nodes in the graph. Such degree-preserving permutation test has been previously used for analyzing enrichment within protein-protein interaction networks [26, 27]. In our tests, when comparing HG to the degree preserving permutation test, we observed that the latter was often much more stringent and produced less results.

### Shortcomings and future plans

Using a data-driven approach, which considers all possible classes, while correcting for multiple tests, is very strict, and thus may increase the rate of false negative findings. In addition, the analysis is conducted under the assumption of specific null models, which, in some cases, may not hold. Other null models can be added to RichMind in the future based on user requests. Another possible future direction for improved large-scale analysis is ranking-based tests, which proved successful in many genomic applications [14].

The use of enrichment analysis is always based on some previously established mapping that is used as an annotation. The selection of a specific annotation system may have a crucial effect on the results of the analysis. For this reason, it would be ideal to use an established functional mapping of the brain that is accepted in the field as “common ground”. Such annotation systems exists in other fields for this type of analysis, e.g. the Gene Ontology initiative [35] or the Kyoto Encyclopedia of Genes and Genomes (KEGG) database [36], which are used as standard gene annotations in computational genomics analysis. However, due to the lack of such a common ground in neuroscience, we adopted a functional annotation that was based on a previously published study, conducted on the 1000 connectomes data, and an anatomic annotation of lobe-laterality information that was based on the TD atlas. We believe that established mapping systems will be available in the near future, and will encourage and improve the use of enrichment analysis in the field. Alternatively, an annotation can be defined based on an independent analysis in order to allow comparing finding across studies or datasets.

Lastly, although RichMind was designed to address the issue of rigorous inference from large-scale findings of a single study, the use of enrichment analysis may potentially be extended into a framework for cross-study meta-analytic inference, as has been demonstrated for genetic data [33]. Such an extension is a possible direction for future work in the field.

### Availability and requirements

RichMind runs on Matlab (version R2011a or later). The software, simulation data and sample data for case study 2 are freely available for download at http://acgt.cs.tau.ac.il/RichMind. The dataset of case study 1 is available from the authors (lauri.nummenmaa@aalto.fi). A technical user manual is available at http://acgt.cs.tau.ac.il/RichMind/help.html.

## Supporting Information

S1 Dataset Dataset for case study 2. (TAR)

S1 Fig An illustration of the difference between the two significance scores in connection group analysis. Each of the graphs contains 20 nodes, of which three are labeled “green” and three are labeled “red”. The number of connections between green nodes and red nodes is 7 in both cases. However, the number of connections and consequently the degree of the nodes varies greatly between the two cases. Graph (a) was found to be enriched with red-green connections using HG-test (FDR q = 4.4*10–5) but not using DPP (FDR q = 0.22). On the other hand, graph (b) was found to be enriched with red-green connections using DPP (FDR q<0.001) but not using HG test (FDR q = 0.34).
(TIF)

S2 Fig Degree distributions on real and simulated networks. The histograms show the degree distribution of the network CC363 analyzed in the study (a), and of instances of scale-free networks of same size randomly generated using power law with exp = 2 (b) and exp = 3 (c).
(TIF)

S3 Fig Simulation results. Graphs with 244 nodes and 363 edges were randomly generated using a power law wiring scheme with exp = 2 (in a,c) and exp = 3 (in b,d), and then complete 5x5 subgraphs were implanted and their edges were randomly removed according to noise level q. The–log of mean p-value over 1000 runs is plotted against level of introduced noise (q parameter). Error bars correspond to one show standard deviation. P-values were estimated by RichMind for the “real” implanted 5X5 bicluster A-B (blue) and for “dummy” 5X5 bicluster A’-B’ (red), using HG test (in a,b) and DPP with 1000 randomized graphs (in c,d).
(TIF)

S4 Fig HG vs. DPP test. Graphs with 244 nodes and 1000 edges were randomly generated using a power law scheme with exp = 2, and then complete bipartite 5x5 subgraphs were planted in them and some of their edges were randomly and independently removed according to noise level q. The–log of mean p-value over 1000 runs is plotted against level of introduced noise (q parameter). Error bars correspond to one show standard deviation. P-values were estimated by RichMind for the “real” implanted 5X5 bicluster A-B (blue) and for “dummy” 5X5 bicluster A’-B’ (red), with A’ and B’ selected to contain high degree nodes. HG test results are shown in a. DPP test results are shown in b. Each DPP test was run with 1000 randomized graphs.
(TIF)

S1 Table RichMind results for case study 1 using 7 functional brain network reported in [15] as annotations; Class abbreviations: VN = visual network, SMN = sensori-motor network, DMN = default-mode network, FPCN = fronto-parietal control network (PDF)

S2 Table RichMind results for case study 2 using 7 functional brain network reported in [15] as annotations; Class abbreviations: VN = visual network, SMN = sensori-motor network, DAN = dorsal attention network, VAN = ventral attention network, DMN = default-mode network (PDF)

S1 Text Method validation supplementary section. (DOCX)
