# Supplementary figures and images for: RichMind: A Tool for Improved Inference from Large-Scale Neuroimaging Results
Source: PLoS One. 2016 Jul 25;11(7):e0159643. doi: 10.1371/journal.pone.0159643 (PMC4959697; doi:10.1371/journal.pone.0159643)

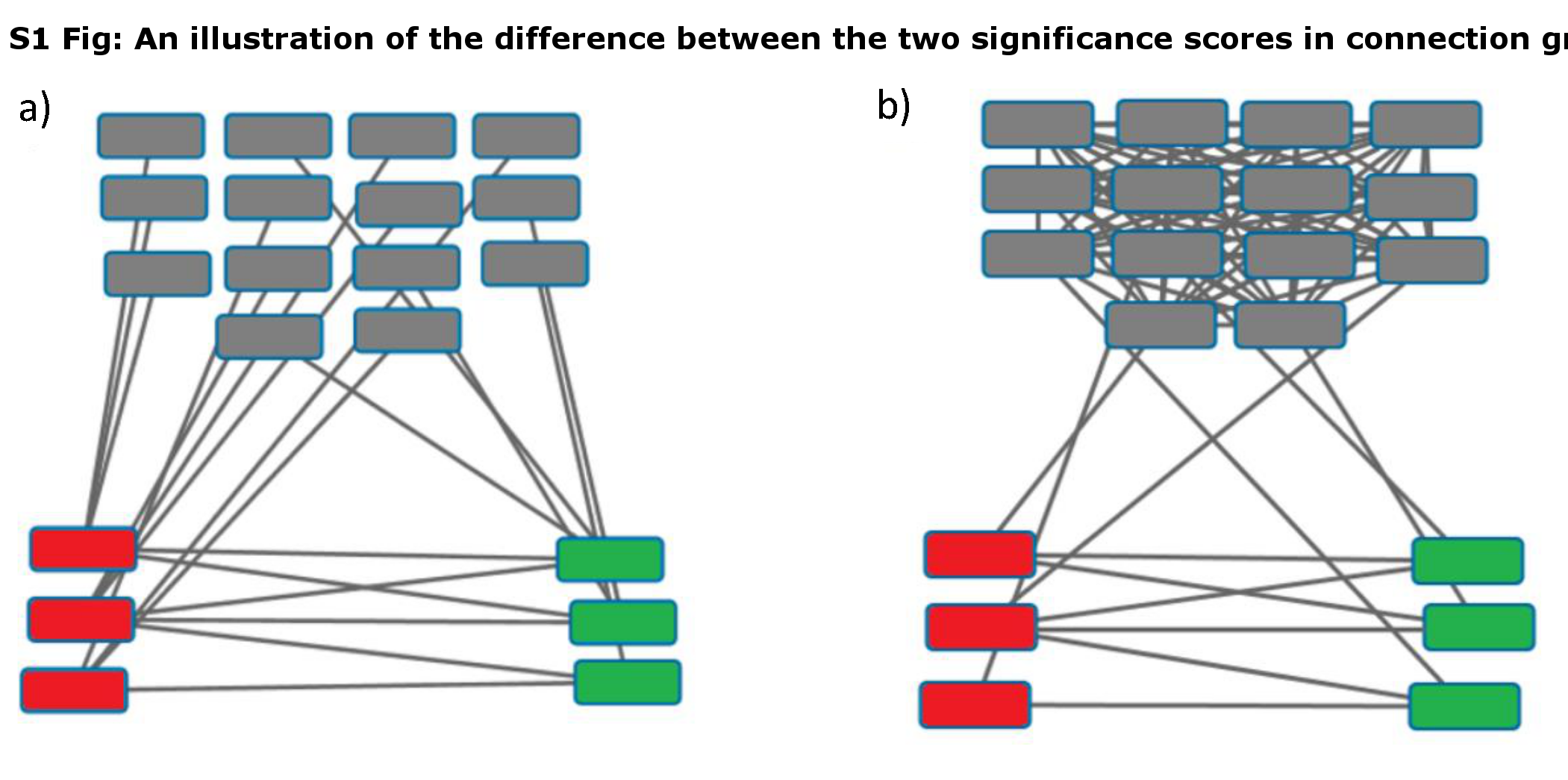

Supplement: S1 Fig — Each of the graphs contains 20 nodes, of which three are labeled “green” and three are labeled “red”. The number of connections between green nodes and red nodes is 7 in both cases. However, the number of connections and consequently the degree of the nodes varies greatly between the two cases. Graph (a) was found to be enriched with red-green connections using HG-test (FDR q = 4.4*10–5) but not using DPP (FDR q = 0.22). On the other hand, graph (b) was found to be enriched with red-green connections using DPP (FDR q<0.001) but not using HG test (FDR q = 0.34). (TIF) [file pone.0159643.s002.tif]

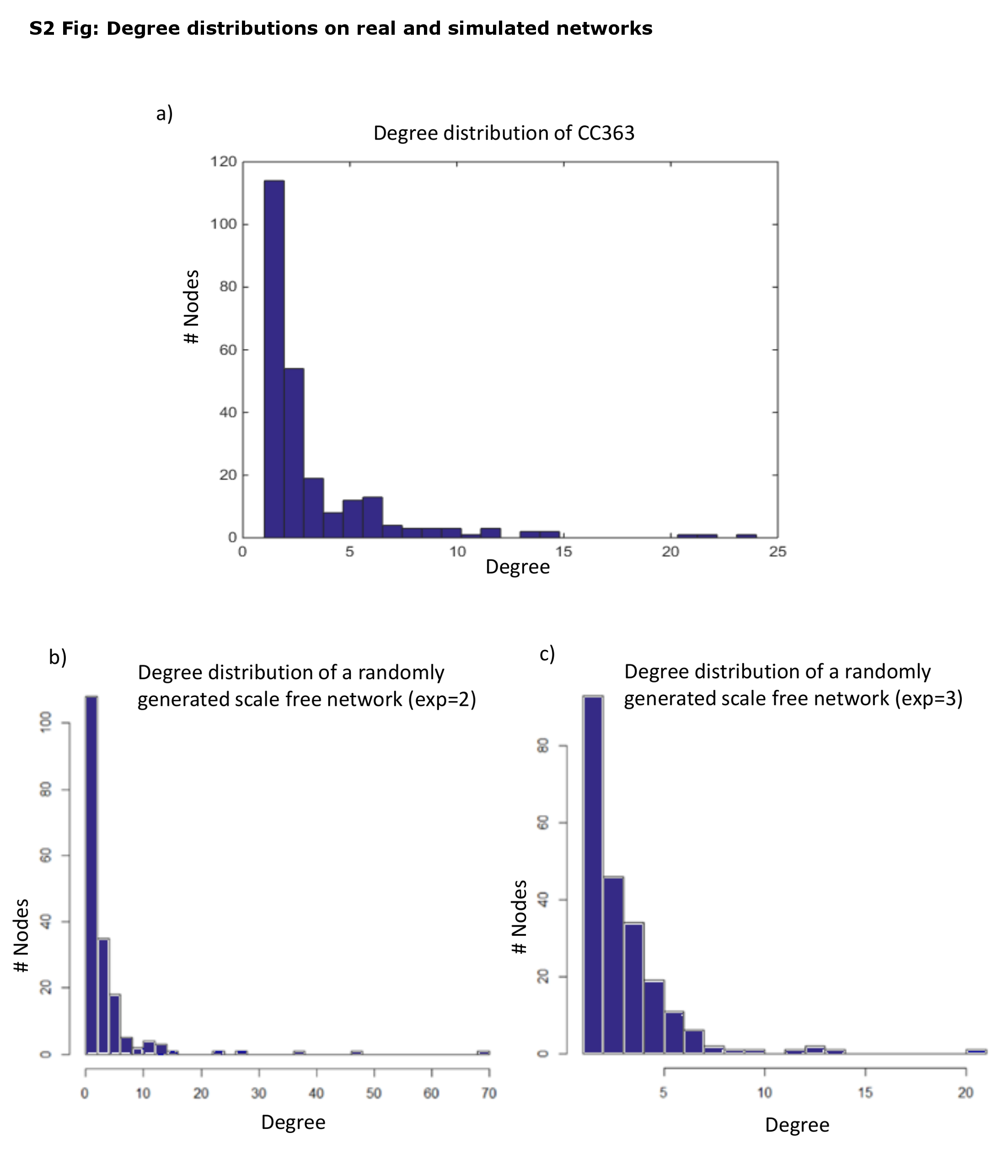

Supplement: S2 Fig — The histograms show the degree distribution of the network CC363 analyzed in the study (a), and of instances of scale-free networks of same size randomly generated using power law with exp = 2 (b) and exp = 3 (c). (TIF) [file pone.0159643.s003.tif]

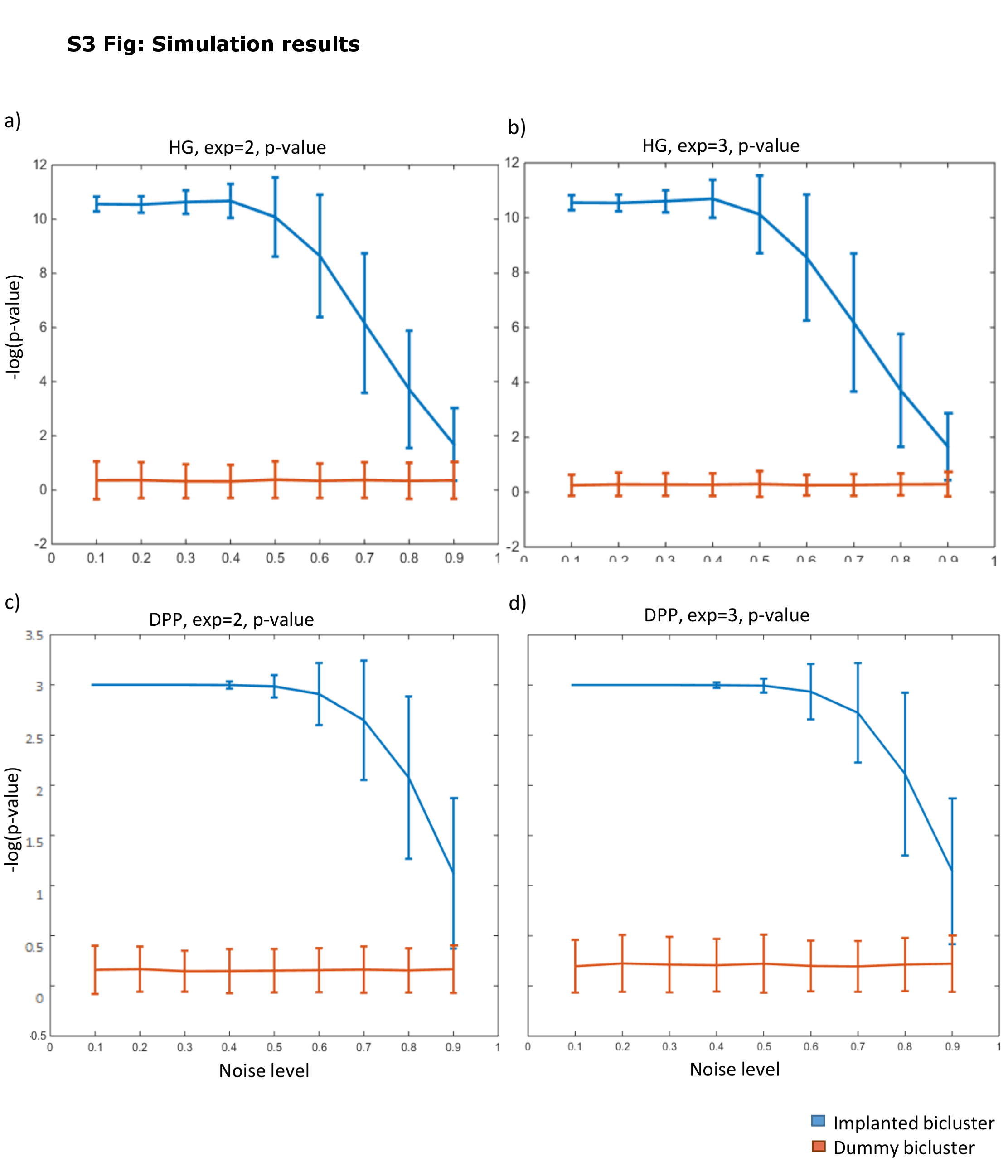

Supplement: S3 Fig — Graphs with 244 nodes and 363 edges were randomly generated using a power law wiring scheme with exp = 2 (in a,c) and exp = 3 (in b,d), and then complete 5x5 subgraphs were implanted and their edges were randomly removed according to noise level q. The–log of mean p-value over 1000 runs is plotted against level of introduced noise (q parameter). Error bars correspond to one show standard deviation. P-values were estimated by RichMind for the “real” implanted 5X5 bicluster A-B (blue) and for “dummy” 5X5 bicluster A’-B’ (red), using HG test (in a,b) and DPP with 1000 randomized graphs (in c,d). (TIF) [file pone.0159643.s004.tif]

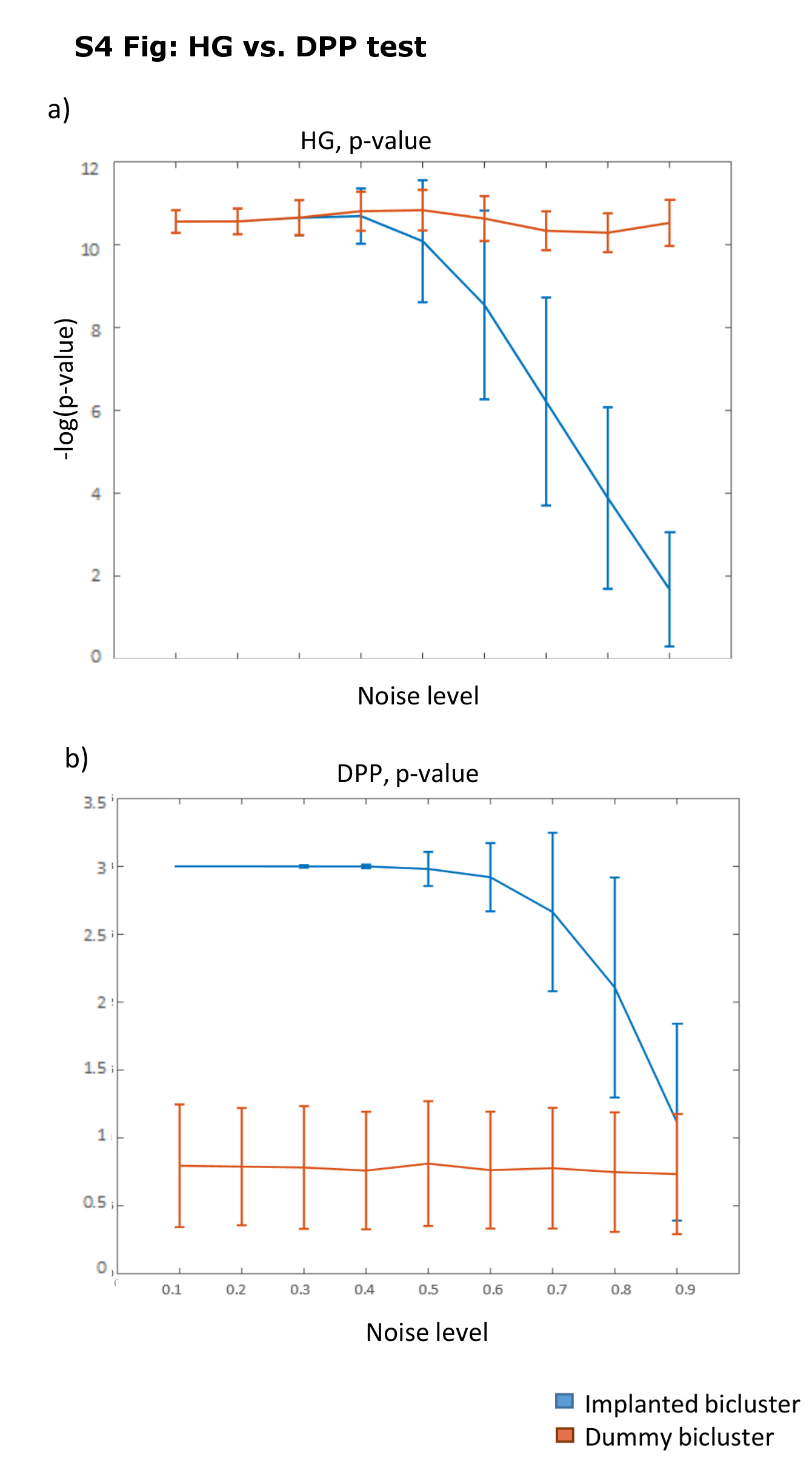

Supplement: S4 Fig — Graphs with 244 nodes and 1000 edges were randomly generated using a power law scheme with exp = 2, and then complete bipartite 5x5 subgraphs were planted in them and some of their edges were randomly and independently removed according to noise level q. The–log of mean p-value over 1000 runs is plotted against level of introduced noise (q parameter). Error bars correspond to one show standard deviation. P-values were estimated by RichMind for the “real” implanted 5X5 bicluster A-B (blue) and for “dummy” 5X5 bicluster A’-B’ (red), with A’ and B’ selected to contain high degree nodes. HG test results are shown in a. DPP test results are shown in b. Each DPP test was run with 1000 randomized graphs. (TIF) [file pone.0159643.s005.tif]
