# Supplementary material for: RichMind: A Tool for Improved Inference from Large-Scale Neuroimaging Results
Source: PLoS One. 2016 Jul 25;11(7):e0159643. doi: 10.1371/journal.pone.0159643 (PMC4959697; doi:10.1371/journal.pone.0159643)
Supplement: S1 Table — (PDF) [file pone.0159643.s006.pdf]

Table S1 : RichMind results for case study 1 using 7 functional brain network reported in [1] as annotations ; Class abbreviations: VN=visual network, SMN=sensori-motor network, DMN=default-mode network, FPCN=fronto-parietal control network

Enriched inter-class connection	HG- based q-value	Permutation based q-value	Frequency ratio	# Underlying connections
VN-VN	2.46E-08	0.3192	4.6958	29
DMN-FPCN	1	<0.0075	0.9225	16
SMN-VN	0	<0.0075	4.0277	49

References

1. Yeo BT, Krienen FM, Sepulcre J, Sabuncu MR, Lashkari D, et al. (2011) The organization of the human cerebral cortex estimated by intrinsic functional connectivity. Journal of Neurophysiology 106: 1125-1165.
