# Supplementary material for: RichMind: A Tool for Improved Inference from Large-Scale Neuroimaging Results
Source: PLoS One. 2016 Jul 25;11(7):e0159643. doi: 10.1371/journal.pone.0159643 (PMC4959697; doi:10.1371/journal.pone.0159643)
Supplement: S2 Table — (PDF) [file pone.0159643.s007.pdf]

Table S2 : RichMind results for case study 2 using 7 functional brain network reported in [1] as annotations ; Class abbreviations: VN=visual network, SMN=sensori-motor network, DAN=dorsal attention network, VAN=ventral attention network, DMN=default-mode network

Set	Enriched class	HG-based q-values	Frequency Ratio	# Underlying voxels
Arousal ISCs	VN	2.34E-08	3.3537	6280
Arousal ISCs	SMN	3.54E-05	1.0911	1843
Arousal ISCs	DAN	2.30E-08	2.9566	3882
Arousal ISCs	VAN	2.38E-08	1.7254	2047
Valence ICSs	DAN	1.02E-08	1.3273	784
Valence ICSs	VAN	2.30E-08	1.8756	1001
Valence ICSs	DMN	2.30E-08	2.5176	2888

References

1. Yeo BT, Krienen FM, Sepulcre J, Sabuncu MR, Lashkari D, et al. (2011) The organization of the human cerebral cortex estimated by intrinsic functional connectivity. Journal of Neurophysiology 106: 1125-1165.
