# Supplementary material for: RichMind: A Tool for Improved Inference from Large-Scale Neuroimaging Results
Source: PLoS One. 2016 Jul 25;11(7):e0159643. doi: 10.1371/journal.pone.0159643 (PMC4959697; doi:10.1371/journal.pone.0159643)
Supplement: S1 Text — (DOCX) [file pone.0159643.s008.docx]

**RichMind: a tool for improved inference from large-scale neuroimaging results**

**Method validation supplementary section**

**The difference between HG and DPP methods**

Fig S1 shows a toy example that illustrates the difference between the two tests that are used for connection-group analysis. Graph (a) is very sparse, and the degree of “red” and “green” nodes is high relative to the rest of the graph. As a result, the p-value for “red”-“green” connectivity within it is significant when calculated using HG, but not when calculated using DPP. In contrast, graph (b) is denser, and the degree of “red” and “green” nodes is relatively low. As a result, the p-value for “red”-“green” connectivity within (b) is significant when calculated using DPP, but not when calculated using HG. The number of connections between “red” and “green” nodes is identical in (a) and (b).

The difference between the two tests emerges from their different assumptions on the nature of “chance” connections (i.e. the “null model”). The HG test assumes that under H_0_ the observed graph was randomly selected from the set$\Sigma$ of all graphs with the same number of edges over the same set of nodes, while the DPP test assumes that under H_0_ the observed graph was randomly selected from the set$\Sigma$ of all graphs with the same set of nodes and node degrees. Consequently, the HG test would favor cases where the number of “red”-“green” connections is high with respect to the overall number of edges in the graph (as is the case in graph (a) but not in graph (b)), while DPP test would favor cases where the number of “red”-“green” connections is high with respect to the number of connections in which “red” or “green” nodes are involved (as is the case in graph (b) but not in graph (a)). The difference between the two methods is further demonstrated using simulated data in S4 Fig.

**RichMind analysis on simulated data**

We generated graphs for which degree distribution follows a power-law (i.e., most nodes have a low degree and a few nodes have a high degree). Such graphs, also called scale-free or small-world graphs, were selected for the simulations as it was previously shown that they have similar properties to networks observed in neuroscience [1-3]. We created graphs with 244 nodes and 363 edges, the same parameters as the CC363 graph of Wang et al. (2013) analyzed in this study. We used the igraph R package [4] to create the graphs. The exponent parameter of the power-law distribution was set to either 2 or 3 as these values produced degree distributions similar to the distribution observed in the real data. S2 Fig shows the degree distributions of the real and simulated networks.

For each simulated graph we planted a 5X5 fully connected bicluster (A, B). That is, we randomly selected two disjoint sets A and B of five nodes each, added all edges between nodes in A and nodes in B, and then added noise to the connections between A and B by removing each edge independently with probability q. This simulation represents the situation where A and B are two annotation classes with more connections between them than expected by chance. In addition, we selected two disjoint random sets A' and B' of five nodes each to represent a “dummy” bicluster (A’,B’), or irrelevant annotations that are not expected to share more connections between them than the background graph. We performed our two tests for connectivity (the HG test, and the non-parametric DPP test) in order to compute a p-value for the two pairs of node sets A-B and A'-B'.

In S3 Fig we show the median and maximal p-values over 10 runs. In each case we tested a wide range of values for q, the noise parameter. We show -log_10_ (p-value) for the connectivity between A and B separately (blue line) and for connectivity between A’ and B’ (red line). The former represents a real group of connections in the graph, whereas the other pairs represent random signals.

Our results show that both the HG test and the DPP test perform very well in detecting the true connections group planted in the data. On the other hand, the p-values of the random “dummy” biclusters are consistently high, which suggests that such results will be rejected as not significant. Also, when we look at the maximal p-value obtained over the different simulation run, it seems that the HG test performs better than the DPP test when the noise levels are very high (e.g., q > 0.5).

The difference between the HG and DPP approaches is further illustrated in S3 Fig, where we repeated the same simulations with denser graphs. The graphs still had 244 nodes, but 1000 edges and exp=2. In this simulation, we deliberately selected A’ and B’ to contain the nodes with the highest degrees that were not in the real bicluster. As can be seen in S4 Fig, such cases are better addressed using the DPP approach.

It should be noted that in our simulations, 11% of these highly connected “dummy” biclusters resulted in p<0.05 (i.e. false positives). This means that the nominal p-values are too liberal to evaluate FDR, and a smaller alpha is needed (in our simulations using alpha=0.02 would give 0.05 FDR). As the DPP test used an MCMC approach in order to sample directly from the set of all graphs with the same degree distribution, our results suggest that simulating power-law graphs represents a harder case, which leads to relatively high percentage of false discoveries. A solution to this problem could be to simulate directly graphs with a power-law distribution instead of our MCMC process. However, this solution will be specific to power-law graphs and not a general solution to any kind of data. Hence, our recommendation for such harder cases is to use a lower significance threshold. Future work can determine which of these null distributions (power law vs. our assumption) better fits fMRI data, and consequently new tests that could be easily integrated into RichMind.

**References**

1. Achard S, Salvador R, Whitcher B, Suckling J, Bullmore E (2006) A resilient, low-frequency, small-world human brain functional network with highly connected association cortical hubs. The Journal of neuroscience 26: 63-72.

2. Bullmore E, Sporns O (2009) Complex brain networks: graph theoretical analysis of structural and functional systems. Nature Reviews Neuroscience 10: 186-198.

3. Bullmore E, Sporns O (2012) The economy of brain network organization. Nature Reviews Neuroscience 13: 336-349.

4. Csardi G, Nepusz T (2006) The igraph software package for complex network research. InterJournal, Complex Systems 1695: 1-9.

5. Yeo BT, Krienen FM, Sepulcre J, Sabuncu MR, Lashkari D, et al. (2011) The organization of the human cerebral cortex estimated by intrinsic functional connectivity. Journal of Neurophysiology 106: 1125-1165.
